# Editorial: Transdiagnostic approaches to child and adolescent mental health—Integrating development, dimensions, and mechanisms

**DOI:** 10.1002/jcv2.70162

**Published:** 2026-09-01

**Authors:** Alessio Bellato, Stephen P. Becker, Catharina A. Hartman, Henrik Larsson, James J. Li, Samantha J. Lynch, Giorgia Michelini

**Affiliations:** ^1^ School of Psychology University of Southampton Southampton UK; ^2^ Developmental Evidence Synthesis, Prediction, Implementation (EPI) Lab Centre for Innovation in Mental Health University of Southampton Southampton UK; ^3^ School of Psychology University of Nottingham Semenyih Malaysia; ^4^ South‐East Asia Mental Health Consortium (SEAMHC) University of Nottingham Semenyih Malaysia; ^5^ Institute for Life Sciences University of Southampton Southampton UK; ^6^ Division of Behavioral Medicine and Clinical Psychology Cincinnati Children’s Hospital Medical Center, Cincinnati Ohio USA; ^7^ Department of Pediatrics University of Cincinnati College of Medicine Cincinnati Ohio USA; ^8^ Universitair Medisch Centrum Groningen Groningen Netherlands; ^9^ Department of Medical Epidemiology and Biostatistics Karolinska Institutet Stockholm Sweden; ^10^ School of Medical Sciences Örebro University Örebro Sweden; ^11^ Department of Psychology University of Wisconsin‐Madison Madison Wisconsin USA; ^12^ Waisman Center University of Wisconsin‐Madison Madison Wisconsin USA; ^13^ Department of Psychiatry and Addiction Faculty of Medicine University of Montreal Montreal Quebec Canada; ^14^ Azrieli Research Center of the Centre Hospitalier Universitaire (CHU) Sainte Justine Mother‐Child University Hospital Montreal Quebec Canada; ^15^ The Matilda Centre for Research in Mental Health and Substance Use University of Sydney Sydney New South Wales Australia; ^16^ Department of Psychology School of Biological and Behavioural Sciences Queen Mary University of London London UK; ^17^ Centre for Brain and Behaviour Queen Mary University of London London UK

**Keywords:** editorial, mental health, practice, research, transdiagnostic

## Abstract

Co‐occurrence of child and adolescent neurodevelopmental and mental health conditions is the rule rather than the exception, yet translating this insight into shared frameworks and clinical practice remains challenging. In this Editorial, we are pleased to introduce the 26 papers included in the 2026 Special Issue of *JCPP Advances*. This Special Issue aimed to advance our understanding of transdiagnostic mechanisms, dimensions, and practices in child and adolescent mental health, and includes original articles, reviews, commentaries, and an editorial perspective. Collectively, these articles illustrate three main ‘transdiagnostic’ conceptualisations; (i) mechanistic approaches identifying shared biological, cognitive, or affective processes across diagnostic boundaries; (ii) dimensional approaches mapping symptom covariance onto hierarchical structures such as the general ‘*p*’ factor and internalising, externalising, and neurodevelopmental spectra; and (iii) developmental, clinical‐staging approaches treating early, non‐specific features as precursors to a broad range of later outcomes. Methodologically, the current Special Issue highlights both the opportunities and limitations of large existing datasets, multi‐informant assessment, and neuroimaging and genomic approaches, while underscoring the persistent difficulty of modelling transdiagnostic dimensions developmentally. Importantly, while translation into routine clinical care remains limited, the field is now well positioned to test the clinical utility and effectiveness of transdiagnostic dimensional assessments and interventions in routine care.

## Key Points

- This editorial introduces the *JCPP Advances* 2026 Special Issue ‘Transdiagnostic Approaches to Child and Adolescent Mental Health’.
- The Issue presents 26 articles including original studies, reviews, commentaries, and an editorial perspective.
- We discuss three main ‘transdiagnostic’ conceptualisations and methods commonly utilised in the literature.

Co‐occurrence of child and adolescent neurodevelopmental and mental health conditions is the rule rather than the exception. Many children referred to mental health services present with complex clinical needs and risk profiles characterised by dimensional variation across multiple diagnostic categories (Zhou et al., 2026). The 2026 Special Issue of *JCPP Advances* was developed to advance our understanding of transdiagnostic mechanisms, dimensions, and practices in child and adolescent mental health. In this Editorial, we reflect on how different ‘transdiagnostic’ conceptualisations could shape research study designs, and consider the barriers and opportunities involved in translating evidence from transdiagnostic research into clinical guidance.

## TRANSDIAGNOSTIC CONCEPTUALISATIONS

Importantly, ‘transdiagnostic’ does not refer to a single approach but a family of related, and at times overlapping, conceptualisations. Some researchers adopt a mechanistic and process‐driven framework (e.g., in line with the Research Domain Criteria, RDoC; Insel et al., 2010) that identifies cross‐cutting biological, cognitive, or affective mechanisms driving dysfunction across traditional diagnostic boundaries. Others focus on a dimensional classification lens (e.g., the Hierarchical Taxonomy of Psychopathology, HiTOP; Kotov et al., 2017) that maps the shared covariance of symptoms into hierarchical dimensions, such as the general ‘*p*’ factor or internalising and externalising spectra. A third approach, aligned with developmental and clinical‐staging perspectives, sees transdiagnostic features such as anxiety, irritability, and low mood as early, non‐specific precursors that predict a broad range of later mental health outcomes (Shah et al., 2020). The 26 papers included in this Special Issue map neatly onto these three lenses.

In line with the first mechanistic lens, Lloyd et al. (2026) used a person‐centred approach in a sample of 559 adolescents at heightened risk of general psychopathology to delineate homogeneous socio‐affective clusters based on task performance and self‐reports. Multiple socio‐affective clusters were identified, some of which were associated with more severe mental health problems that aligned with traditional diagnostic boundaries, whereas others showed broader transdiagnostic associations with internalising problems. Other studies sought to generate mechanistic insights by leveraging neuroimaging data, such as functional magnetic resonance imaging and electroencephalography (EEG). For example, using data from the Adolescent Brain Cognitive Development (ABCD) study, Devine et al. (2026) found specific alterations in functional connectivity within and between networks involved in self‐focused thought, attention, and cognitive control, which might contribute to the development of general psychopathology in early adolescence, as also recently suggested by a large transdiagnostic mega‐analysis within the ENIGMA consortium (Townend et al., 2026). In a prospective longitudinal cohort, Valdes et al. (2026) further found that early left frontal alpha asymmetry measured using EEG at 12 months predicted social attention, empathy, and social approach at age 2. Lower levels of social attention emerged as the key pathway linking early brain function to higher levels of ADHD, autism, and depression symptoms.

In line with the second transdiagnostic approach, multiple studies focussed on well‐established dimensions of psychopathology, such as internalising and externalising spectra (Carrington et al., 2026), while also adding new evidence on emerging dimensions, such as the neurodevelopmental spectrum (Michelini et al., 2024). For example, Peng and Watts (2026), using longitudinal data from almost 12,000 children, showed that ADHD bridges both externalising and neurodevelopmental domains, highlighting the value of using dimensional approaches for parsing heterogeneity in diagnostic categories and patterns of comorbidity. This study offers an elegant test of the alternative common cause and dynamic mutualism models of psychiatric co‐occurrence. A similar approach could be extended to other dimensions, including the general psychopathology (‘*p*’) factor, as suggested in a commentary accompanying this issue (Wang et al., 2026). Other studies further applied latent variable modelling approaches to GWAS data to validate psychopathology dimensions at the aetiological level. For example, using genomic structural equation modelling of GWAS data on intellectual and neurodevelopmental conditions (i.e., ADHD, autism, Tourette's, intellectual disability, language disorders), Morgan et al. (2026) showed that neurodevelopmental and intellectual disabilities represent separable dimensions, at least at the genomic level. By applying a similar approach to GWAS data from more than 3 million participants, Keser et al. (2026) found that the overlap between 11 neurodevelopmental and psychiatric disorders could largely be attributed to transdiagnostic genetic liability, which could correspond to the *p* factor commonly identified using phenotypic data.

A final set of studies illustrate innovative applications of the clinical‐staging approach. Häfeli et al. (2026) used data from 1186 Swiss adolescents from the general population and observed that non‐specific traits, such as worry, body dissatisfaction and weight concerns, predicted multiple psychopathological traits (particularly anxiety and depression) and shaped symptom trajectories over time. Additionally, Kim et al. (2026) and Rinne et al. (2026) documented caregiver transdiagnostic symptoms—parental anxiety and maternal depression, respectively—and the intergenerational associations with mental health traits and diagnoses in their offspring. Lastly, Marinca et al. (2026) investigated the associations between self‐reported sleep problems, teacher‐reported behavioural symptoms (hyperactivity/impulsivity, inattention, conduct) and self‐reported emotional symptoms (anxiety, depression) in over 800 children living in East London. They used network analysis and identified seven distinct symptom clusters—some purely behavioural, others reflecting mixed sleep–emotional symptoms or sleep problems alone—with sleep and bedtime‐related anxiety emerging as central, transdiagnostic nodes within the network.

## TRANSDIAGNOSTIC METHODOLOGIES

Besides addressing transdiagnostic research through different lenses, the studies included in this Special Issue implemented different methodologies, each carrying its own strengths and limitations. Most studies relied on large datasets, whether drawing on open samples such as the ABCD study (Devine et al., 2026; Martinez et al., 2026; Peng & Watts, 2026), electronic health records (Altschuler et al., 2026), or new school‐based samples (Marinca et al., 2026; Černis et al., 2026). Large datasets afford the statistical power needed to detect small transdiagnostic effects that more modestly sized samples may likely miss, and they allow dimensional constructs to be modelled across a breadth of symptoms and disorders. Electronic health records and school‐based datasets further offer a degree of ecological validity and reach into populations that traditionally referred samples struggle to capture. Building on this, the use of existing cohorts such as the ABCD study also permits transdiagnostic questions to be addressed longitudinally, yielding insights into developmental processes. However, gaps in coverage of available assessments—particularly for less commonly measured domains—can shape which transdiagnostic questions get asked, and which do not, irrespective of their theoretical and clinical importance.

Another important challenge concerns the informants on whom these datasets depend. Self‐, parent‐/caregiver‐ and teacher‐reports frequently diverge, particularly in adolescence, when parents and caregivers typically have progressively less direct knowledge of their children’s internal states and day‐to‐day behaviour, as young people become less inclined to disclose emotional difficulties to caregivers and spend more time in activities away from home. As highlighted by Martinez et al. (2026), these discrepancies in youth symptom ratings across reporters and development are not simply noise—they may themselves carry meaningful information about context‐specific symptom presentation. For example, youth in the ABCD study consistently rated their own symptoms higher than caregivers and teachers did, with discrepancies widening over time. Importantly, such discrepancies were systematically impacted by youth characteristics (e.g., larger for females and non‐White youth) as well as caregiver and social factors, highlighting the value of multi‐informant studies particularly in the context of diverse socio‐cultural settings.

Another methodological challenge pertains to the difficulty of modelling latent dimensions longitudinally—without assuming that either the dimension itself, or the indicators used to measure it, remain constant over time. This is, in our view, one of the greatest barriers to progress in the application of transdiagnostic approaches to child and adolescent mental health, requiring appropriate study designs and statistical modelling approaches to accommodate changing sets of indicators across development and changes in manifestation from early childhood through adolescence. Only then will frameworks such as HiTOP and RDoC move beyond a mostly static snapshot towards genuinely developmental accounts. Building on this, greater investment in cross‐cultural and diverse samples, particularly from low‐ and middle‐income countries, is similarly overdue, given that current evidence of transdiagnostic constructs remains largely confined to majority White, Western populations.

## FUTURE DIRECTIONS

While transdiagnostic research in child and adolescent mental health has matured beyond initial proof‐of‐concept, its translation into routine clinical care remains limited. As highlighted by Chetcuti et al. (2026) in this Special Issue, this may be because dimensional or transdiagnostic approaches can complicate distinguishing clinically meaningful differences from age‐normative developmental variation, which in turn limits the provision of appropriate support. The implementation of transdiagnostic approaches to clinical assessment is also hindered by the fact that most, though not all, assessment tools are designed to capture categorical rather than dimensional constructs. Future research should aim to optimise existing instruments or develop new ones capable of capturing clinically significant dimensions without overburdening health services.

To bridge this translation gap, future research must shift from describing and modelling transdiagnostic dimensions to explicitly testing the effectiveness of using transdiagnostic approaches to clinical assessment and treatment in clinical settings. For example, pragmatic clinical trials should systematically assess the benefits of using transdiagnostic assessments and interventions over approaches based on categorical diagnoses. It would also be important for the next generation of studies to examine both shared (transdiagnostic) and unique (syndrome‐specific) features within the same study, rather than treating shared processes in isolation. Scaling this work will require integrating multidisciplinary tools—such as ecological momentary assessment, computational modelling, and neuroimaging—across fine‐grained developmental time scales, alongside low‐burden approaches like passive data collection for real‐world clinical settings. Ultimately, this will reveal how these processes unfold across multiple time scales, from moment‐to‐moment fluctuations to year‐to‐year developmental trajectories, to inform personalised, mechanism‐focused interventions. We hope the papers in this Special Issue will help bridge different conceptualisations of transdiagnostic research and stimulate the next generation of studies in this field.

## AUTHOR CONTRIBUTIONS

**Alessio Bellato**: Conceptualization; writing—original draft; writing—review and editing. **Stephen P. Becker**: Writing—review and editing. **Catharina A. Hartman**: Writing—review and editing. **Henrik Larsson**: Writing—review and editing. **James J. Li**: Writing—review and editing. **Samantha J. Lynch**: Writing—review and editing. **Giorgia Michelini**: Conceptualization, writing—review and editing.

## CONFLICT OF INTEREST STATEMENT

Alessio Bellato declares: (a) funding from the National Institute for Health and Care Research (NIHR208319) and the Academy of Medical Sciences (NGR2\1430), (b) honoraria from the Association for Child and Adolescent Mental Health (ACAMH) for educational activities, (c) honoraria as Joint Editor of *JCPP Advances*, (d) consultancy honoraria from the Cyprus Research and Innovation Foundation, Swiss National Science Foundation, and Wallenberg Foundation, and (e) travel reimbursements from the ACAMH and the International Brain Research Organization (IBRO); none of this related to the present project. He is also a former member of the NHS England ADHD taskforce. Stephen P. Becker reports research grant support from the National Institutes of Health (R01MH122415, R01MH138336, R01MH139504), the Institute of Education Sciences, U.S. Department of Education (R324A240155, R305A240106), the Spencer Foundation, and the Cincinnati Children's Research Foundation. Stephen P. Becker reports receiving royalties for a book from Guilford Press and for continuing education courses from Premier Educational Seminars, Inc. (PESI) and J&K Seminars, and honorarium as Joint Editor of *JCPP Advances*. Henrik Larsson reports receiving grants from TAKEDA; personal fees from and serving as a speaker for Medice, Takeda Pharmaceuticals and Neuraxpharm Sweden AB; advisory board for TAKEDA and Neuraxpharm Sweden AB; all outside the submitted work. Henrik Larsson is editor‐in‐chief of *JCPP Advances*. Catharina A. Hartman is the Deputy Editor of *JCPP Advances*. Giorgia Michelini reports research grant support from Medical Research Council grant UKRI1506 and honoraria from the Association for Child and Adolescent Mental Health (ACAMH) for educational activities and as Joint Editor of *JCPP Advances*. All other authors have no conflicts of interest or financial interests to declare.

## ETHICAL CONSIDERATIONS

Ethical approval is not applicable for this Editorial article.

## Data Availability

Data sharing not applicable to this article as no datasets were generated or analysed for this Editorial article.

## References

[jcv270162-bib-0001] Altschuler, M. R. , Tong, X. , Shah, P. , Verona, E. , Bowling, J. M. , Krueger, R. F. , Kotov, R. , McAllister, J. H. , Voit, M. , Paudel, S. , Leon, S. C. , Kala, S. , & Lyons, J. S. (2026). Positive developmental cascades: Strength development reduces support needs in children. JCPP Advances, e70097. 10.1002/jcv2.70097 42416638 PMC13338963

[jcv270162-bib-0002] Carrington, S. J. , Keating, J. , Uljarević, M. , Abbot‐Smith, K. , Jones, C. R. G. , & Leekam, S. R. (2026). Understanding the associations between neurodevelopmental features and internalising and externalising behaviours: A transdiagnostic approach. JCPP Advances, e70110. 10.1002/jcv2.70110 42416637 PMC13338984

[jcv270162-bib-0003] Černis, E. , Antonović, M. , Lofthouse, K. , Shipp, M. L. , & Waite, P. (2026). ‘Felt sense of anomaly’‐type transdiagnostic dissociative experiences in adolescents: Endorsed phenomenology and plausible mechanisms. JCPP Advances, e70116. 10.1002/jcv2.70116 42416669 PMC13339579

[jcv270162-bib-0004] Chetcuti, L. , Spackman, E. , Begeer, S. , Scheeren, A. M. , Alvares, G. A. , Trembath, D. , Whitehouse, A. J. O. , Trollor, J. N. , Hedley, D. , Haslam, N. , McPartland, J. C. , Krueger, R. F. , & Uljarević, M. (2026). Barriers and pathways to adopting transdiagnostic dimensional approaches in pediatric research and clinical care. JCPP Advances, e70145. 10.1002/jcv2.70145

[jcv270162-bib-0005] Devine, J. J. , Hosterman, G. R. , Sloss, J. , & Romer, A. L. (2026). Alterations in resting‐state functional connectivity relate to psychopathology trajectories during emerging adolescence. JCPP Advances, e70135. 10.1002/jcv2.70135 42416655 PMC13339399

[jcv270162-bib-0006] Häfeli, X. A. , Morosan, L. , Hirsig, A. , & Schmidt, S. J. (2026). Transdiagnostic symptom networks in adolescent psychopathology: A longitudinal panel network analysis. JCPP Advances, e70118. 10.1002/jcv2.70118 42416623 PMC13339512

[jcv270162-bib-0007] Insel, T. , Cuthbert, B. , Garvey, M. , Heinssen, R. , Pine, D. S. , Quinn, K. , Sanislow, C. , & Wang, P. (2010). Research domain criteria (RDoC): Toward a new classification framework for research on mental disorders. American Journal of Psychiatry, 167(7), 748–751. 10.1176/appi.ajp.2010.09091379 20595427

[jcv270162-bib-0008] Keser, E. , Liao, W. , Allegrini, A. G. , Rimfeld, K. , Eley, T. C. , Plomin, R. , & Malanchini, M. (2026). Isolating transdiagnostic effects reveals specific genetic profiles in psychiatric disorders. JCPP Advances, e70129. 10.1002/jcv2.70129 42416636 PMC13338967

[jcv270162-bib-0009] Kim, H. , Pan, L. , Talati, A. , Gameroff, M. J. , Weissman, M. M. , & Simpson, H. B. (2026). Intergenerational transmission of anxiety: A comparison of transdiagnostic and disorder‐specific pathways. JCPP Advances, e70117. 10.1002/jcv2.70117 42416668 PMC13339481

[jcv270162-bib-0010] Kotov, R. , Krueger, R. F. , Watson, D. , Achenbach, T. M. , Althoff, R. R. , Bagby, R. M. , Brown, T. A. , Carpenter, W. T. , Caspi, A. , Clark, L. A. , Eaton, N. R. , Forbes, M. K. , Forbush, K. T. , Goldberg, D. , Hasin, D. , Hyman, S. E. , Ivanova, M. Y. , Lynam, D. R. , Markon, K. , …, & Zimmerman, M. (2017). The hierarchical taxonomy of psychopathology (HiTOP): A dimensional alternative to traditional nosologies. Journal of Abnormal Psychology, 126(4), 454–477. 10.1037/abn0000258 28333488

[jcv270162-bib-0011] Lloyd, A. , Astle, D. , Wu, T. (C. H.) , Steinbeis, N. , Chokhani, R. , Lucas, L. , Fearon, P. , & Viding, E. (2026). Transdiagnostic profiles of socio‐affective functioning in adolescents at‐risk of poor mental health. JCPP Advances, e70137. 10.1002/jcv2.70137 42416631 PMC13339588

[jcv270162-bib-0012] Marinca, A. A. , Michalek, J. E. , Gregory, A. M. , Ali, A. , & Lau, J. Y. F. (2026). Sleep disturbance as a transdiagnostic marker of children’s mental health difficulties: A network analysis of item‐level associations between different types of sleep problems and different behavioural and emotional symptoms. JCPP Advances, e70104. 10.1002/jcv2.70104 42416653 PMC13339339

[jcv270162-bib-0013] Martinez, M. , Mittal, V. A. , Schaefer, J. D. , Haase, C. M. , & Damme, K. S. F. (2026). Diverging perspectives on emerging mental health symptoms: Multi‐informant discrepancies and their associated determinants across the transition to adolescence in the Adolescent Brain Cognitive Development Study. JCPP Advances, e70122. 10.1002/jcv2.70122 42416656 PMC13339503

[jcv270162-bib-0014] Michelini, G. , Carlisi, C. O. , Eaton, N. R. , Elison, J. T. , Haltigan, J. D. , Kotov, R. , Krueger, R. F. , Latzman, R. D. , Li, J. J. , Levin‐Aspenson, H. F. , Salum, G. A. , South, S. C. , Stanton, K. , Waldman, I. D. , & Wilson, S. (2024). Where do neurodevelopmental conditions fit in transdiagnostic psychiatric frameworks? Incorporating a new neurodevelopmental spectrum. World Psychiatry, 23(3), 333–357. 10.1002/wps.21225 39279404 PMC11403200

[jcv270162-bib-0015] Morgan, M. J. , Malanchini, M. , Robinson, E. B. , & Ronald, A. (2026). Multivariate genetic analyses test for the presence of a general ‘*n*’ factor underlying neurodevelopmental conditions. JCPP Advances, e70168. 10.1002/jcv2.70168

[jcv270162-bib-0016] Peng, Z. , & Watts, A. L. (2026). Common cause versus dynamic mutualism: Insights into ADHD’s common comorbidities. JCPP Advances, e70142. 10.1002/jcv2.70142 42416609 PMC13339281

[jcv270162-bib-0017] Rinne, G. R. , Berryhill, B. L. , Chandler, C. M. , & Somers, J. A. (2026). Maternal depression across childhood and offspring young adult depression and anxiety: Testing adolescent emotion dynamics as transdiagnostic mechanisms. JCPP Advances, e70130. 10.1002/jcv2.70130 42416672 PMC13339266

[jcv270162-bib-0018] Shah, J. L. , Scott, J. , McGorry, P. D. , Cross, S. P. M. , Keshavan, M. S. , Nelson, B. , Wood, S. J. , Marwaha, S. , Yung, A. R. , Scott, E. M. , Öngür, D. , Conus, P. , Henry, C. , Hickie, I. B. , & the International Working Group on Transdiagnostic Clinical Staging in Youth Mental Health . (2020). Transdiagnostic clinical staging in youth mental health: A first international consensus statement. World Psychiatry, 19(2), 233–242. 10.1002/wps.20745 32394576 PMC7215079

[jcv270162-bib-0019] Townend, S. , Staginnus, M. , Gao, Y. , Alexander, N. , Arolt, V. , Banaschewski, T. , Beesdo‐Baum, K. , Bellgrove, M. A. , Benegal, V. , Blair, R. J. , Blanco‐Hinojo, L. , Boeken, O. J. , Böhnlein, J. , Bölte, S. , Bonnekoh, L. M. , Brandeis, D. , Bressan, R. A. , Breuer, F. , Bruin, W. B. , …, & Fairchild, G. (2026). Shared and distinct alterations in brain structure of youth with internalizing or externalizing disorders: Findings from the ENIGMA antisocial behavior, ADHD, major depressive disorder, and anxiety working groups. Biological Psychiatry, 100(4), 399–413. 10.1016/j.biopsych.2025.08.003 40812743

[jcv270162-bib-0020] Valdes, V. , Lutri, N. , Chung, H. , & Nelson, C. A. (2026). Infant frontal alpha asymmetry predicts social attention and transdiagnostic risk for emotional reactivity. JCPP Advances, e70089. 10.1002/jcv2.70089 42416614 PMC13339033

[jcv270162-bib-0021] Wang, L.‐L. , Wang, Yi. , & Chan, R. C. K. (2026). Commentary: Toward an Integrated Understanding of General Psychopathology—A commentary on Devine et al. (2026). JCPP Advances, e70163. 10.1002/jcv2.70163 42633013 PMC13498740

[jcv270162-bib-0022] Zhou, X. , Jia, F. , Bambling, M. , Edirippulige, S. , Whiteford, H. , & Lin, J. (2025). Associations between multiple neurodevelopmental disorders and mental health in children. Journal of Affective Disorders, 393, 120397. 10.1016/j.jad.2025.120397 41072876

